# Exploring the genetics of nestling personality traits in a wild passerine bird: testing the phenotypic gambit

**DOI:** 10.1002/ece3.412

**Published:** 2012-11-02

**Authors:** Jon E Brommer, Edward Kluen

**Affiliations:** 1 Department of Biology, University of Turku, University HillFI-20014 Turku, Finland; 2 ARONIA Coastal Zone Research Team, Novia University of Applied Sciences and Åbo Akademi UniversityRaseborgsvägen 9, FI-10600 Ekenäs, Finland; 3 Bird Ecology Unit, Department of Biosciences, University of HelsinkiP.O. Box 65 (Viikinkaari 1), FI-00014 Helsinki, Finland

**Keywords:** Aggression, animal personality, bird, cross foster, genetic correlation, quantitative genetics, wild population

## Abstract

When several personality traits covary, they form a behavioral syndrome. Understanding the evolutionary dynamics of a behavioral syndrome requires knowledge of its genetic underpinning. At present, our understanding of the genetic basis of behavioral syndromes is largely restricted to domestic and laboratory animals. Wild behavioral syndromes are mostly inferred on the basis of phenotypic correlations, and thus make the “phenotypic gambit” of assuming that these phenotypic correlations capture the underlying genetic correlations. On the basis of 3 years of reciprocal cross-fostering of 2896 nestlings of 271 families within a pedigreed population, we show that the nestling personality traits handling aggression, breathing rate, and docility are heritable (*h*^2^ = 16–29%), and often have a pronounced “nest-of-rearing” variance component (10–15%), but a relatively small “nest-of-origin” variance component (0–7%). The three nestling personality traits form a behavioral syndrome on the phenotypic and genetic level. Overall, the phenotypic correlations provide a satisfactory description of the genetic ones, but significantly underestimate the magnitude of one of the pairwise genetic correlations, which mirrors the conclusion based on domestic and laboratory studies.

## Introduction

Quantitative genetics is a statistical approach aiming to understand the genetic architecture of traits and their evolutionary dynamics (Fisher 1958; Falconer and MacKay 1996; Lynch & Walsh 1998). Behavioral ecologists are increasingly turning to quantitative genetic concepts in an effort to understand the evolutionary premises under which variation in animal personality subsists in wild populations (Sih et al. 2004a; Réale et al. 2007, 2010; Dochtermann and Roff 2010). Animals often display consistent behavior when exposed to mildly stressful conditions, for example, in terms of their aggression. Such a consistent behavior is termed an animal personality trait or temperamental trait (Réale et al. 2007). Multiple personality traits often covary; for example, aggression and boldness may covary (reviewed in Koolhaas et al. 1999). When the correlation between behavioral traits are maintained across situations, these traits are said to form a behavioral syndrome (Sih et al. 2004a,b). For example, aggressive individuals are also bold, both when a predator is present and when predation risk is low. Correlated personalities are found in the wild. For example, aggression in western sunbirds is genetically correlated with dispersal (Duckworth and Kruuk 2009). The existence of animal personality and behavioral syndromes in the wild is often considered puzzling, because classical theory in behavioral ecology is based on individuals optimizing their behavior to their environment in a facultative manner (Krebs and Davies 1978), which would imply selection favoring individual flexibility in behavior (Sih et al. 2004a,b). However, individual behaviors are often relatively inflexible (average repeatability of 37%, Bell et al. 2009). One explanation of why we observe that individual behavior is repeatable is that genetic effects are underlying it, where the heritability of a behavioral trait sets the lower expectation for repeatability (Falconer and MacKay 1996). By extension, the reason that several personality traits show a high intraindividual correlation may be that each of these traits have a heritable component and that these components are genetically correlated. In general, a genetic correlation can arise because of pleiotropic effects, where the same genes affect more than one behavior, or different genes code for the personality traits, but are in linkage disequilibrium because a selective force is maintaining specific combinations of coding these genes (Falconer and MacKay 1996; Lynch and Walsh 1998). Nevertheless, we know little about the genetics of personality traits (e.g., van Oers et al. 2005; Réale et al. 2007; Dochtermann and Roff 2010), and especially genetic correlations between personality traits are poorly studied (Dochtermann 2011; van Oers and Sinn 2011).

One insight from quantitative genetics with clear relevance for the study of animal personality concerns what has been termed the “phenotypic gambit” (Grafen 1984; cf. Krebs and Davies 1978) or “Cheverud's conjecture” (Cheverud 1988). Suppose X and Y are two behavioral traits (e.g., aggression and boldness). We may then observe a phenotypic covariation between these two traits, but we cannot be sure that this covariation is really based on intrinsic (i.e., heritable) properties. A phenotypic trait value can be decomposed (in its simplest formulation) into a genetic or breeding value and an environmental value (Falconer and MacKay 1996; Lynch and Walsh 1998). We can then write out the phenotypic correlation *r*_P_ as the average of the weighted sum of genetic correlation *r*_G_ and the environmental correlation *r*_E_, each weighted by the geometric mean of the fraction of variance, which is heritable (heritability) and not heritable, respectively. That is (Roff 1997),


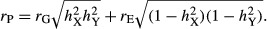
(1)

An environmental correlation between traits may arise because good environmental (i.e., nonheritable) conditions allow individuals to be both aggressive and bold. Clearly, under natural conditions, such environmental correlations may readily arise. The phenotypic gambit is then to assume that the phenotypic correlation *r*_P_ between traits still largely reflects the underlying genetic correlation *r*_G_. This gambit is made whenever invoking evolutionary arguments on the basis of phenotypic correlations. This is because only the genetic correlation can play a role in evolutionary considerations as covariances based on environmental conditions are transient and not inherited by the next generation (Falconer and MacKay 1996; Lynch and Walsh 1998). From a life-history perspective, it has been pointed out that a negative genetic correlation between traits (a trade-off) can be phenotypically masked by a strong positive environmental correlation (van Noordwijk and de Jong 1986) and clearly such trade-offs may also apply to personality traits.

Much of the literature on animal behavioral syndromes is based on phenotypic correlations, thereby explicitly or implicitly making the phenotypic gambit (Dochtermann 2011; van Oers and Sinn 2011). For highly heritable traits, such as morphometrical traits, the phenotypic correlation will resemble the genetic correlation (Roff 2007), because the geometric mean heritability of traits X and Y 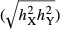 will exceed the geometric mean of their nonheritable proportions of phenotypic variances 
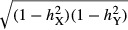
. Personality traits have, however, a modest heritability (e.g., Réale et al. 2007; van Oers and Sinn 2011), of around 0.3 or lower. This implies that the observed phenotypic correlation between personality traits is largely determined by the magnitude of the nongenetic correlation *r*_E_. Indeed, based on published estimates of phenotypic and genetic correlations of behavior, Dochtermann (2011) concluded that the sign of phenotypic and genetic correlations agreed, but that the magnitude of the genetic correlation between personality traits was not necessarily captured well by its phenotypic correlation. Based on this recent review, it is apparent, that (1) few estimates of phenotypic and genetic correlations in personality traits are available (data on 13 species, Dochtermann 2011) and that (2) most of the studies included by Dochtermann (2011) were based on domestic animals or were performed in the laboratory. In order to allow more robust generalization on whether the phenotypic gambit holds in personality research, we clearly need more information, especially from wild populations.

In this study, we explore the genetic basis of three simple behavioral measures taken from blue tit *Cyanistes caeruleus* nestlings prior to fledging. We focus on nestlings, because this allows us to reciprocally cross-foster broods for 3 consecutive years within a pedigreed population. This design allows us to partition the phenotypic (co)variances into additive genetic, nest-of-origin, nest-of-rearing, and residual components. Because we work with nestlings, we are restricted to take simple field-based behavioral measures of how the nestling respond to the stress of being handled in terms of their docility during a fixed time period, their breathing rate (cf. Fucikova et al. 2009; Naguib et al. 2011) and their overall handling aggression. First, we aim to establish the genetic versus other sources of variance on these simple offspring behavioral assays taken in the wild. We consider those behavioral traits, which indeed have a genetic basis to qualify as personality traits. This is because the presence of heritable variation indicates significant between-individual variance and therefore consistency in these behavioral traits. Our second aim is to test whether phenotypic covariances between nestling personality traits indeed reflect the genetic correlations. That is, can we make the phenotypic gambit when studying correlated personality traits in the wild?

## Material and Methods

Blue tits were studied in south-western Finland in a study area of approximately 10-km^2^ in size, situated on the north-east side of the city of Tammisaari (60°01′N, 23°31′E). All birds included in this study bred in nest boxes, which were made available for breeding starting in 2003. Laying dates and clutch sizes were established by weekly checking all nest boxes. The clutchsize of blue tits in this population is typically 8–14 eggs. Hatching date (day 0) of at least the first chick in a brood was established by daily hatch checks carried out in the afternoon starting at 1 day prior to the day of expected hatching (see Kluen et al. 2011 for details). Hatchlings were jointly weighed to estimate the average mass at hatching. Adults were caught caring for young and were assumed to be the nestlings' social parents. Adult birds were sexed on the basis of the presence or absence of a brood patch. When not ringed as offspring, adult individuals were ringed to allow lifelong identification. All offspring were ringed in 2005. From 2006 onwards, offspring and parents were ringed. Data on nestling behavioral traits in this study were collected from 2007 up to and including 2009.

### Reciprocal cross-fostering and sexing of nestlings

In this population, the last egg(s) regularly hatch 2 days after the first one. Our procedure of reciprocal cross-fostering was primarily designed to swap an equal number of nestlings of the same age and the same mass between two nests. Nests where the oldest nestlings were 2 days (day 2) were paired, primarily on the basis of the average mass of hatchlings encountered at day 0 and, when possible, with respect to their brood size. Our interest was in separating genetic from environmental effects, and differences in brood size between paired nests can from this viewpoint be considered part of the differences in the environment experienced by the nestlings. Controlling for discrepancies in body mass at cross-fostering between nestlings is important for this design, because even small discrepancies can readily translate into developmental differences later in life. An equal number of nestlings were reciprocally swapped between two nests. The pair of families between which nestlings were swapped, we here term “dyad,” and the brood in which a nestling hatched was termed “nest of origin” and the one in which it was reared “nest of rearing.” The decision on which nestlings were swapped was made random-systematically. In the first nest of a dyad, nestlings were weighed and individually marked by clipping a unique combination of their toe nails. By the toss of a coin, it was decided whether the heaviest nestling stayed in its nest of origin or was moved to another nest of rearing. Staying or moving was then alternated down the mass hierarchy in the nest. The same procedure was conducted in the second nest in a dyad if its brood size allowed doing so. In case of large differences in brood size between nests in a dyad, the nest with the smallest brood size was visited first and the number of nestlings swapped was thus approximately half the brood size of the nest with the smallest brood size. Nestlings of equal body mass were then selected from the second nest with the larger brood size (size-matched). In some cases, nestlings in nests with similar average mass of hatchlings had already diverged in their mass during the two pre-crossfoster days, in which case, cross-fostering was not carried out. Some families could not be part of the cross-fostering protocol, either because there was no other brood with nestlings of similar mass or because an odd number of nests hatched on the same day. Cross-fostering was carried out on first broods only. In 2007 and 2008, collection of data on behavioral traits overlapped with an experiment where the ectoparasite load of nests were reduced by taking the original nest cup from the nest box at cross-fostering (day 2) and replacing it by a nest cup that was cleaned of ectoparasites by microwaving (see Pitala et al. 2009 for details). This manipulation has a mild effect on the morphological development of the nestlings (Pitala et al. 2009; Brommer et al. 2011) and may affect nestling behavioral measures. Offspring were ringed when the oldest nestling was 9 days old with a metal ring at which age the toe-nail clippings is still clearly visible and each marked 2-day-old nestling thus remained identifiable during its lifetime.

On day 9, two to five feathers were sampled from the back of the nestling and stored in 95% ethanol. DNA was extracted from one small feather using the protocol of Elphinstone et al. (2003). Sexing was based on a polymerase chain reaction (PCR) with sex-chromosome specific primers (P2 and P8; Griffiths et al. 1998) using GE Healthcare “ready-to-go” PCR beads following the manufacturer's instructions. The product was visualized on an agarose gel stained with ethidium bromide. Nestlings for which amplification was not successful after two separate extraction and PCR trials were considered of “unknown” sex.

### Quantifying offspring behaviors

When the oldest nestlings were 16 days old, all nestlings were taken from the nest box and put individually in a numbered small paper bag (100 × 235 × 40 mm, Pro-Pac, Sydney Australia). Once every nestling was placed in its own paper bag, all bags were reshuffled in order to randomize the order of further handling the nestling from the order in which nestlings were individually placed in the paper bags. Bags were picked one-by-one to carry out the following behavioral measurements.

1. *Docility:* The nestling was immediately placed with its back on the palm of the observer's hand, held with its neck between the observer's index and middle finger, with one leg held by the foot between thumb and index finger and the other by the middle and ring finger. Once secured, the nestling was held still at approximately 40-cm from the observer's face. A stopwatch was started and the number of struggles during a fixed time period (20 sec in 2007, 10 sec in later years) were counted. Docility was expressed as −1 times the number of struggles per second, such that a higher docility score indicated an individual which was more docile (i.e., struggled less). This test has analogs in production animals (e.g., Hessing et al. 1993).
2. *Breathing rate:* Holding the nestling in the same position as described above, the observer then used the lap function of the stopwatch to, with minimal delay after the time period stated above, time how long it took the offspring to take 30 breaths. This timing was, without delay, repeated a second time. Breathing rate is calculated by taking the average of these two measurements and expressing it as the number of breaths per second. Breathing rates have been quantified also in great tits, where it is assumed to reflect the degree by which a bird is stressed by the handling (Carere and van Oers 2004). In great tit nestlings, a measure of handling stress based on breathing rates correlated with exploratory behavior (Fucikova et al. 2009). Note, however, that the breathing rate assay used in great tit nestlings (Fucikova et al. 2009; Naguib et al. 2011) is different from ours in that it focuses on the change in breathing rate during four assays taken in 1 min, whereas our assay quantifies the nestling's breathing rate shortly after the start of handling the individual.
3. *Handling aggression:* Morphological measurements were then taken in the following order (1) tarsus length (using a sliding caliper), which was measured twice, (2) head length from the tip of the beak to the back of the skull (using a sliding caliper), (3) wing length (with a ruler), (4) tail length (with a ruler). Handling aggression is a Likert-scale score (Likert 1932) describing the nestling's aggressive response (struggling, picking) during the taking of these measurements, ranging from 1 (completely passive during all measurements) to 5 (struggling during the whole time it takes to perform the measurements). Measuring tarsus was the most uncomfortable procedure for most nestlings, as it involves tightly holding the tarsometatarsus in a low angle to the tibiotarsus and folding the foot inward to be consistent with the tarsometatarsus. A handling aggression score of 3 can be considered as typical, where the nestling shows aggression during measurement of the tarsus and at one later measurement. Handling aggression thus reflects when a nestling “calmed down” while handling it in different positions during approximately 5 min. The handling aggression scoring procedure does not take into account the behavior of the nestling during the docility assay or when counting breathing rates. A similar scoring procedure has been used in other studies, for example in a wild population of bighorn sheep (Réale et al. 2000).

Being in isolation from its nest mates in the paper bag is likely to be stressful for offspring. We therefore considered the order in which the nestlings were measured as a potentially important covariate, because it reflects the duration a nestling has been in isolation in its paper bag and thus, potentially, the amount of stress it experienced prior to measuring. All measurements were taken by either EK or JEB.

### Animal model analysis: general

Analyses were performed in a mixed model framework where information on the relatedness between individuals was used to estimate the additive genetic effects (animal model, e.g., Lynch and Walsh 1998). As we were interested in the genetic and nongenetic relationship between traits, we constructed models, where the uni or multivariate **G** matrix was estimated by defining the linear mixed model


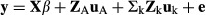
(2)

where **y** is a vector of observations on all individuals, β is a vector of one or more fixed effects, **X** represents a design matrix (of 0s and 1s) relating the appropriate fixed effects to each individual, **u**_A_ is a vector of additive genetic (random) effects, with **Z**_A_ the design matrix relating the appropriate additive genetic effects to each individual. The summation Σ_k_**Z**_k_**u**_k_ allows for additional random effect structures on the individual and **e** is a vector of residual errors. **G** is defined as the matrix for vector **u**_A_ and its elements (the genetic (co)variances) can be estimated by using information on the coefficient of coancestry *Θ*_*ij*_ between individuals *i* and *j*, which is directly obtained from the pedigree. The additive genetic effects for trait *t* were assumed to be normally distributed with mean of zero (i.e., defined relative to the trait-specific fixed-effect mean) and with an additive genetic variance of *σ*^*2*^_A,*t*_. This variance (and the additive genetic covariance between all traits considered) was estimated using Restricted Maximum Likelihood (REML) from the variance-covariance matrix of additive genetic effects, which is equal to **A***σ*^*2*^, where **A** has elements *A*_*ij*_ = *2Θ*_*ij*_. The models were implemented in ASReml 3 (VSN International, Hemel Hempstead, U.K.) and solved under REML.

We used the social pedigree, where all offspring in one family are assumed to be full-sibs. The proportion of extra-pair nestlings in this population was not known, but population-specific data of nine populations suggests that between 7% and 25% of our blue tit nestlings could have been sired by an extra-pair male (Brommer et al. 2010). Because the maternal links are not affected by extra-pair paternity, it has been shown in blue tits that quantitative genetic estimates are robust to this relatively low rate of extra-pair paternities (Charmantier and Réale 2005). We used a pruned pedigree, where only the individuals with phenotypic measures are retained and their ancestors. This pedigree covered a maximum of five generation and listed 2461 individuals with 205 sires (50 sires of sire, 54 dams of sire) and 237 dams (42 sire of dam, 46 dams of dam).

### Animal model analysis of nestlings

We initially considered each behavioral measure separately in order to test which fixed and random effects were important. The fixed effect structure of equation (2) accounted for year, nestling's sex, observer (EK, JEB), and measurement order (as a factor with 14 levels). In addition, we investigated the potential fixed effect of early environmental covariates, including the mass of the nestling at day 2 (prior to cross-fostering) standardized to zero mean, as well as a factorial variable coding whether the nestling was moved to another nest (cross-fostered, coded as 1) or whether it was reared in its native nest (coded as 0).

Variation after taking into account, the above stated fixed effects was partitioned into additive genetic, nest-of-origin, nest-of-rearing, and residual variance components. Estimation of the additive genetic (co)variances is based on the resemblance of population-wide relatives. In our design, the “nest of origin” variance refers to variance across broods of presumed full-sibs that occur in addition to the variance across broods based on their breeding value (additive genetic variance). Simulations show that cross-fostering within a pedigreed population is an approach which, when analyzed in an animal model context, allows to separate nest of origin from additive genetic variances (Kruuk and Hadfield 2007). Nest-of-origin variance may be caused by several nonexclusive factors (Kruuk et al. 2001; Kruuk and Hadfield 2007). (1) Early common-environmental effects which nestlings experienced prior to cross-fostering during incubation and the 2 days together in their nest of origin (including variation in brood size). (2) Effects the different mothers may have on their offspring (maternal effect). (3) Nonadditive genetic (dominance) variance. Estimation of dominance variance is challenging in the wild, but animal model analysis of captive animals with similar data structure suggest that dominance variance will not end up in the estimate for additive genetic variance, but on the level of the “brood” (Serenius et al. 2006) or in the residuals (Kruuk 2004; Adams et al. 2012). Our design does not allow us to partition this variance further. In particular, we have too few repeated records of mothers to estimate the maternal variance (results not shown).

Nest-of-rearing variation captures the common-environmental effect shared by all offspring in their nest of rearing during nestling ontogeny from day 2–16. This includes such varied aspects as variation in brood size across nests, differential parental (male plus female) capacities in rearing the offspring under their care. Furthermore, environmental characteristics of the nest box of rearing (e.g., microclimate) and local environmental conditions, such as food supply, will affect nest of rearing variance.

We viewed the random effects for “nest of origin” and “nest of rearing” as variables related to our experimental design and therefore did not formally test their statistical significance. The additive genetic effect was tested by carrying out a Likelihood Ratio Test (LRT), where –two times the difference in log-likelihood between a model, including the additive genetic effect and a model without this effect was tested against a chi-square distribution with one degree of freedom. Fixed effects were tested using a Wald *F*-tests with the residual degree of freedom numerically derived following Kenward and Roger (1997).

### Multi-variate model and the phenotypic gambit

For the multivariate version of equation (2), we kept the same random effect structure and included those fixed effects which were significant in the univariate model. Hence, we obtained (co)variance matrices for residual, nest-of-origin, nest-of-rearing, and additive genetic effects. Correlations were calculated following the standard definition of a correlation with their standard error obtained by applying the delta method (Lynch and Walsh 1998). Significance of the difference between the estimated genetic correlation and zero was calculated by a LRT between the unconstrained multivariate model and one where the focal genetic correlation was constrained to zero. We refer to this test as LRT (*r*_A_ = 0).

We first derived the REML phenotypic correlations from the REML phenotypic (co)variance matrix, which can be obtained by summing up all the (co)variance components in the above described multivariate model or, equivalently, from a multivariate mixed model with the fixed effects and residuals. We term these here REML phenotypic correlations to clarify they are estimated under REML, but they are approximately equivalent to the Pearson correlation one would obtain after correcting the raw data for those fixed effects, we included in our model. The REML phenotypic correlation is based on all data and thus has much smaller uncertainty than the genetic correlations, which are based on comparing relatives and thus deal with a subset of all the data. A conservative test of the phenotypic gambit is thus to make a comparison of model likelihood when REML phenotypic correlations are interpreted as genetic ones. Formally, this was based on a LRT between the unconstrained multivariate model and one where the three genetic correlations were constrained to be equal to the REML phenotypic ones (the LRT has thus three degrees of freedom, one for each genetic correlation which in the constrained model are not estimated). In addition, we also tested using LRT whether each of the pairwise (trait–trait) genetic correlation was statistically equivalent to its REML phenotypic equivalent. Because one parameter is constrained, this test has one degree of freedom. We refer to LRT tests where genetic correlation(s) are constrained to be equal to the REML phenotypic one(s) as LRT (*r*_A_ = *r*_P_). In general, there are several ways to compare phenotypic and additive genetic matrices and their various properties (Roff 2007; Dochtermann and Roff 2010). We here consider a full matrix comparison to be outside our present focal interest. Instead, we view the putative equivalence of phenotypic and genetic correlations as the prime hypothesis, which can be tested within a likelihood framework as outlined above.

## Results

### Data on nestling behavioral traits

A total of 2896 nestlings were assayed (except for the docility of 43 nestlings). These nestlings originated from 271 broods, and were reared in 238 nests (for 33 broods one of the nests in the cross-foster dyad perished prior to assaying the nestlings). Average body mass of those nestlings, which were swapped between nests did not differ between the nests (0.0293 g difference, 95% CI of difference: [−0.0244 g, 0.0830 g]) and this difference was only a small fraction (1.6%) of the mean body mass of a 2-day old nestling (1.85 g). We therefore believe that our cross-foster protocol adequately avoided alteration of the composition of broods.

**Figure 1:**
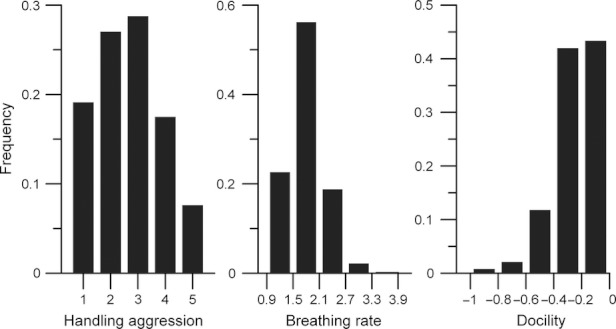
Frequency distributions for the three blue tit nestling personality traits quantified in this study. Handling aggression (*n* = 2896) is a score of 1 (low aggression) to 5 (high aggression) and the frequency of each score is plotted. Breathing rate (*n* = 2896) is expressed in breaths/sec, and docility (*n* = 2853) is expressed in −1 × number of struggles/sec such that high docility values indicate a more docile individual. For these latter two variables, the labels on the *X*-axis give the minimum and maximal values of each bin in the histogram.

The frequencies of handling aggression scores and of breathing rates showed clear unimodal distributions, but the distribution of docility was truncated, where the modal nestling was docile, with <2 struggles during the 10 sec assay period (Fig. 1). Handling aggression varied from 1 to 5 (mean ± SD: 2.67 ± 1.190). Breathing rate varied from 0.92 to 3.9 breaths/sec (mean ± SD: 1.81 ± 0.397) and docility varied from −1 to 0 struggle/sec (mean ± SD: −0.198 ± 0.165).

### Univariate analysis of nestling behavioral measures

All the nestling behavioral measures had a significant heritable component (Table 1). Heritability was modest, ranging from 15.9% to 28.5%. Rearing effects were present for all traits, explaining 10.1% to 15.9% of the total variance. There was no detectable nest-of-origin variance for handling aggression, but variance in breathing rate and docility could be partially attributed (7.3% and 4.0%, respectively) to effects of the nestlings” nest-of-origin on nestling behavior at day 16. All models included the (significant) effect of an individual”s mass at cross-fostering (day 2). Hence, we find that early ontogenetic differences between the nestlings have consequences for their behavior measured 14 days later. Significant annual differences were found for breathing rate and docility (Table 1). For docility, the coefficient of 2008 and 2009 were lower compared to 2007 and this difference may at least be partly due to the changes we made in our assay of docility after 2007 (10s assay instead of 20s), although other differences associated with the year may clearly also have played a role. The cross-fostering procedure itself had no effect on any of the behavioral traits. The measurement order was only important in explaining handling aggression, where nestlings measured later (and which thus spent more time in isolation) reacted more aggressively during handling than those measured early on (Fig. S1). Handling aggression and breathing rate, but not docility, were sexual dimorphic; female nestlings were less aggressive during handling and had a higher breathing rate than male nestlings (Table 1). Differences between the two observers were statistically significant in the measurements of breathing rate and docility (but not for handling aggression), illustrating that – despite the standardized protocol – subtle differences remain between observers in how a nestling responded to being held or in the observers' counting of struggles and breaths.

**Table 1:** Univariate animal model analyses of the three offspring personality traits based on reciprocal cross-fostering over multiple generations

*Trait*/ Type	Source	Estimate ± SE	Proportion (SE)	Test	*P*
***Aggression***	REML phenotypic	1.269 ± 0.0484			
	Residual	0.730 ± 0.0516	0.575 ± 0.0476		
Random	Nest-of-origin^1^	0	0		
Random	Nest-of-rearing	0.177 ± 0.0328	0.140 ± 0.0241		
Random	Genetic	0.361 ± 0.0746	0.285 ± 0.0540	χ^2^ = 16.6	<0.001
Fixed	**Intercept**	2.626 ± 0.208		*F*_1,208.0_ = 2751.5	<0.001
Fixed	Year			*F*_2,243.4_ = 1.05	0.35
Fixed	**Sex**			*F*_2,1939.0_ = 5.60	<0.001
	Male	0.134 ± 0.0453			
	Unknown	0.123 ± 0.130			
Fixed	Observer	−0.130 ± 0.0791		*F*_1,271.1_ = 2.95	0.089
Fixed	**Measure order**			*F*_13,1819.7_ = 10.71	<0.001
Fixed	**Mass at day 2 (g)**	0.647 ± 0.0647		*F*_1,2025.3_ = 100.1	<0.001
Fixed	Cross-fostered	0.0164 ± 0.0440		*F*_1,1872.3_ = 0.91	0.34
***Breathrate***	REML phenotypic	0.138 ± 0.00537			
	Residual	0.0826 ± 0.00642	0.597 ± 0.0517		
Random	Nest-of-origin	0.0101 ± 0.00526	0.0730 ± 0.0379		
Random	Nest-of-rearing	0.0221 ± 0.00040	0.159 ± 0.0267		
Random	Genetic	0.0236 ± 0.0108	0.171 ± 0.0763	χ^2^ = 4.44	0.035
Fixed	**Intercept**	2.011 ± 0.0352		*F*_1,159.6_ = 12107.5	<0.001
Fixed	**Year**			*F*_2,221.5_ = 35.78	<0.001
	2008	−0.00904 ± 0.0369			
	2009	0.252 ± 0.0355			
Fixed	**Sex**			*F*_2,1924.5_ = 6.27	0.002
	Male	−0.0466 ± 0.0145			
	Unknown	−0.0304 ± 0.0420			
Fixed	**Observer**	0.0766 ± 0.0271		*F*_1,283.2_ = 8.01	0.005
Fixed	Measure order			*F*_13,1811.1_ = 0.85	0.61
Fixed	**Mass at day 2 (g)**	−0.128 ± 0.0211		*F*_1,2025.1_ = 32.67	<0.001
Fixed	Cross-fostered	−0.0135 ± 0.0142		*F*_1,1855.5_ = 2.00	0.16
***Docility***	REML phenotypic	0.0244 ± 8.73E−4			
	Residual	0.0171 ± 0.00108	0.699 ± 0.0476		
Random	Nest-of-origin	9.77E−4 ± 7.90E−4	0.0400 ± 0.0323		
Random	Nest-of-rearing	2.47E−3 ± 5.85E−4	0.101 ± 0.0229		
Random	Genetic	3.88E−3 ± 1.66E−2	0.159 ± 0.0664	χ^2^ = 6.82	0.009
Fixed	**Intercept**	−0.247 ± 0.0144		*F*_1,140.8_ = 961.1	<0.001
Fixed	**Year**			*F*_2,193.5_ = 14.75	<0.001
	2008	−0.0548 ± 0.0140			
	2009	−0.0591 ± 0.0135			
Fixed	**Sex**			*F*_2,1927.9_ = 1.19	0.307
	Male	−0.00815 ± 0.0066			
	Unknown	0.00164 ± 0.019			
Fixed	**Observer**	0.0534 ± 0.0104		*F*_1,266.6_ = 26.70	<0.001
Fixed	Measure order			*F*_13,1803.5_ = 1.53	0.10
Fixed	**Mass at day 2 (g)**	−0.0702 ± 0.00092		*F*_1,1848.8_ = 57.65	<0.001
Fixed	Cross-fostered	0.00219 ± 0.0064		*F*_1,1829.1_ = 0.01	0.94

For each trait, all the random and fixed effects included in the mixed model are presented. The estimated variance as well as the proportion of the REML phenotypic variance is given for the residuals and the three random effects, where “Nest-of-origin” specifies the variance due to factors prior to cross-fostering, “Nest-of-rearing” the variance due to the nest in which an individual was reared and “Genetic” the additive genetic effect. The proportion of REML phenotypic variance due to additive genetic effects gives the trait's heritability *h*^2^, the statistical significance of which is tested using a Likelihood Ratio Test. Nestling sex is reported as a contrast to “female” and the category “unknown” relates to the small number of nestlings, which could not be sexed. Mass at day 2 was standardized to zero mean prior to analysis, and has units grammes (g). “Cross-fostered” tests whether those nestlings which were fostered in another nest were different from those who were reared in their natal nest. Fixed effects were tested using an unconditional *F*-test where the residual degrees of freedom were numerically estimated. Significant fixed effects are indicated in bold. Raw data phenotypic SD is reported in the text.

1 Constrained to zero, because negative when left unconstrained.

### Relationship between nestlings' behavioral traits

We constructed a multivariate animal model with the same fixed and random effect structure as the univariate models. Correlations between the behavioral traits were remarkably consistent in sign across all components of variance considered (Table 2, full covariance matrices reported in Table S1). The **G** matrix revealed significant negative correlations between handling aggression and breathing rate and between handling aggression and docility (Table 2). That is, nestlings, which carry genes for more aggressive behaviors are genetically predisposed to breathe slower under handling stress and to also be less docile. In addition, we find that there is a tendency (*P* = 0.098) for genetically more docile individuals to also breath faster. The estimates of the correlations were similar for the different components (residual, genetic, nest of origin, nest of rearing), although the genetic correlations typically were stronger than the correlations for the other components (Table 2).

**Table 2:** Correlations between offspring personality traits handling aggression, breathing rate, and docility for different components of variance

Component/Trait	Breathing rate	Docility
REML phenotypic		
Aggression	−0.278 ± 0.025	−0.463 ± 0.020
Breathing rate		0.372 ± 0.023
Residual		
Aggression	−0.236 ± 0.044	−0.376 ± 0.037
Breathing rate		0.402 ± 0.041
Nest-of-origin		
Aggression	n.e.	n.e.
Breathing rate		−1.63E−4 ± 1.2E−3
Nest-of-rearing		
Aggression	−0.138 ± 0.12	−0.458 ± 0.11
Breathing rate		0.286 ± 0.13
Additive genetic		
Aggression	−0.503 ± 0.15^1^	−0.747 ± 0.12^2^
Breathing rate		0.429 ± 0.21^3^

LRT, Likelihood Ratio Test; REML, Restricted Maximum Likelihood. The full (co)variance matrix is provided in Table S1. REML phenotypic correlations are based on the sum of all (co)variance components. Because the nest-of-origin variance component for handling aggression was constrained to zero (Table 1), correlations with this trait are not estimable (n.e.). For the genetic correlations, we used a LRT to establish the probability the genetic correlation was equal to zero, reported as LRT (*r*_A_ = 0) with one degree of freedom.

1 LRT (*r*_A_ = 0): χ^2^ = 8.8, *P* = 0.0030.

2 LRT (*r*_A_ = 0): χ^2^ = 21.1, *P* < 0.001.

3 LRT (*r*_A_ = 0): χ^2^ = 2.74, *P* = 0.098.

### Comparing phenotypic and genetic correlations

Constraining all three genetic correlations to have the same value as the REML phenotypic ones produced a nonsignificant change in likelihood (LRT (*r*_A_ = *r*_P_): χ^2^ = 5.10, df = 3, *P* = 0.16) indicating that phenotypic correlations provided a reasonable description of the genetic correlations between the three behavioral traits. Nevertheless, pairwise testing suggested a statistical difference for the genetic and REML phenotypic correlation between aggression and docility (LRT (*r*_A_ = *r*_P_): χ^2^ = 4.10, df = 1, *P* = 0.043), which was the strongest genetic correlation (Table 2). The genetic correlations for aggression – breathing rate and breathing rate – docility were statistically equal to the phenotypic ones (LRT (*r*_A_ = *r*_P_): χ^2^ = 2.04, df = 1, *P* = 0.15 and χ^2^ = 0.06, df = 1, *P* = 0.81, respectively).

## Discussion

We performed behavioral assays on blue tit nestlings at a time when they have completed most of their morphological development. We focus on the quantification of three behavioral traits; aggression during handling, breathing rate, and docility, which are readily assayed under field conditions. Based on an experimental design where broods were reciprocally cross-fostered for 3 years and use of quantitative genetic methods, we find that these three traits have a modest, but clearly significant heritable component. This means that these behavioral measures indeed represent an intrinsic property of individuals and therefore describe an individual's personality and not merely reflect within-individual random variance. Additive genetic variance contributed approximately 16–29% of their phenotypic variance. The three personality traits correlate. We find that nestlings, which breathe fast and thus are presumably more stressed by the procedure (Carere and van Oers 2004; Fucikova et al. 2009) are more docile and less aggressive during handling. Because of our design, we can break down these phenotypic correlations into their additive genetic and other components and we here demonstrate that this behavioral syndrome indeed is also found on the additive genetic level. There are significant negative genetic correlations between handling aggression and docility and between aggression and breathing rate and a clear tendency (*P* = 0.098) for a positive genetic correlation between breathing rate and docility. We interpret this finding as evidence that blue tit nestling genotypes differ in their sensitivity to stress. Under the stress of being outside the nest box (in isolation from their siblings) and of being handled, “easily-stressed” genotypes take fast, shallow breaths, and “freeze,” thereby becoming more docile and less aggressive in their response to being handled compared to less easily stressed genotypes.

We can here only speculate about the mechanism by which the genetic correlation between our nestling personality traits could arise. We note, however, that our findings are consistent with all behaviors capturing pleiotropically acting variation in some physiological response to stress. For example, stress leads to hormone secretion by the adrenal system (e.g., corticosteroids), which affect oxygen uptake (breathing rate) and behavioral responses (Silverin 1986, 1998; Cockrem 2007). In particular, corticosterone titers are genetically associated with measures of personality (Martins et al. 2007; Baugh et al. 2012). Although pleiotropy through the actions of the endocrine system is a parsimonious explanation of the correlated personality traits we here describe, we of course cannot exclude that the genes underlying our nestling personality traits are in linkage disequilibrium because of some other process. For instance, males and females may mate disassortatively with respect to nestling docility and handling aggression.

### Early environmental and rearing effects on nestling personality traits

Repeating reciprocal cross-fostering during several years is a powerful method for describing additive genetic parameters (Kruuk and Hadfield 2007). This is because (1) offspring of broods produced in different years by the same parent(s) will be cross-fostered, allowing estimation of the resemblance of full-sibs (in case both parents reproduce together in multiple years) or half-sibs (in case only one parent is the same in multiple years). (2) Some of the assayed offspring will produce offspring themselves, which will be cross-fostered and assayed. The resemblance between all these relatives of varying degrees are comprehensively analyzed in an animal model framework (e.g., Lynch and Walsh 1998; Kruuk and Hadfield 2007). Our experimental design allows separating early environmental (i.e., pre cross-fostering “nest-of origin”) effects from rearing (i.e., post cross-fostering “nest-of-rearing”) effects. Without reciprocal cross-fostering, these two sources of variance are grouped in one common-environmental “brood” variance component (Kruuk and Hadfield 2007). Because the “nest-of-origin” variance concerns resemblance among full-siblings, which occurs additionally to the additive genetic variance, it also includes, among other sources of variance, the nonadditive genetic (dominance) variance (e.g., Serenius et al. 2006). Thus, the “nest-of-origin” variance presents the maximal possible contribution dominance variance makes to the phenotypic variance. Estimation of dominance variance in personality traits is evasive (van Oers et al. 2004) and the extent of this source of variance in personality traits remains largely unknown (van Oers et al. 2005; van Oers and Sinn 2011). In general, however, dominance variation may comprise a relatively large part of the total (i.e., additive and nonadditive) genetic variance in personality. This is because personality traits are likely to be under selection and such traits tend to show a relatively high proportion of dominance variance (Crnokrak and Roff 1995; Roff 2007). Alternatively, the “nest-of-origin” variance component can be interpreted as the maximal contribution females can have on phenotypic variance (via maternal effects). We find relatively little “nest-of-origin” variance in handling aggression and docility, explaining 0%, and 4% of the phenotypic variance, respectively. Nevertheless, “nest of origin” variance contributed 7% of the phenotypic variance in breathing rate, illustrating that nonadditive genetic and/or other sources of early environmental variance can make a clear (i.e., >5%) contribution to the phenotypic variance in a nestling personality trait.

A sizeable contribution to phenotypic variance, when compared to additive genetic effects, is made by the “nest-of-rearing” variance, which explains approximately 16% of phenotypic variance in breathing rates (compared to *h*^2^ = 17%), 10% of docility (*h*^2^ = 16%), and 14% of the variance in handling aggression (*h*^2^ = 28%). Although we here cannot establish the mechanism by which rearing-environmental effects are mediated, these effect sizes do demonstrate that ecological factors, including the social interactions between sibs, may have a considerable impact on a nestling's personality (cf. Naguib et al. 2011). It also seems plausible that the offsprings' parents have, through rearing effects, a sizeable influence on their nestlings' personality. This finding is not surprising, because also morphological traits, such as nestling tarsus length and body mass are strongly affected by rearing effects (Kruuk et al. 2001; Merilä et al. 2001). Nevertheless, elucidating which ecological factors (e.g., food supply, microclimate, parental effects) modify personality traits through rearing effects may be challenging as the variance components, we here identify present the combination of potentially a large number of factors, where each factor by itself may have only a small effect. Within the context of animal personality, an intriguing possibility is that the capacity to rear offspring is dependent on the parents' personality, for example because parents with a certain combination of personality traits perform better (Both et al. 2005; Schuett et al. 2011).

Reciprocal cross-fostering, where part of one family's offspring is fostered in another family and vice versa, is a commonly used method to obtain a first estimate of the genetic component in a trait. This is because in this design, an upper estimate of heritability can be estimated as twice the proportion of the nest-of-origin variance over the phenotypic variance (Falconer and MacKay 1996). The estimate concerns the maximal heritability possible, because it necessarily assumes that all nest-of-origin variance is indeed additive genetic variance and this assumption is unlikely to hold (e.g., Kruuk and Hadfield 2007). Our study thereby provides an indication of how reasonable it is to make this assumption in studying nestling behavioral traits. This is because our design is based on sequential reciprocal cross-fostering within a pedigree population, where we are able to separate these two variance components. We here find low nest-of-origin variances in all three nestling personality traits, forming 0% (handling aggression), 30% (breathing rate), and 20% (docility) of the summed nest-of-origin and additive genetic variance components. Rerunning the models and ignoring the pedigree structure (and thus analyzing the data as a traditional cross-foster design) shows that such traditional analysis indeed agrees in terms of the heritability of handling aggression, but tends to overestimate the heritability of breathing rate and docility, although not significantly so (Table S2). Hence, our findings imply that such traditional cross-fostering techniques could provide a reasonable first-line of evidence for heritable nestling personality traits. In general, however, cross-fostering over multiple generations and use of proper animal models is preferred because traditional cross-fostering is likely to overestimate trait heritability (Kruuk and Hadfield 2007), sometimes dramatically so (Pitala et al. 2007).

### The phenotypic gambit in blue tit nestling personality traits

Our design allows us to evaluate the validity of the phenotypic gambit by partitioning the phenotypic covariance matrix into its underlying components, including the additive genetic covariance matrix and other relevant components. There are many ways in which matrices can be compared (Roff 2007), but we here focus on the phenotypic gambit and ask whether phenotypic correlations provide a statistically reasonable description of the genetic correlations. For the correlated nestling personality traits as a whole, we find that the phenotypic correlation matrix indeed is a sufficient approximation. A striking finding is that the correlations for essentially all variance components are in the same direction and of roughly the same strength. However, pairwise investigation of the genetic correlations underlying the behavioral syndrome shows that the strong genetic correlation between handling aggression and docility was not captured sufficiently by the phenotypic correlation. The majority of phenotypic variance in handling aggression (57.5%) and docility (69.9%) are due to residual effects and the correlation on the level of the residuals (−0.38) thus largely determines the phenotypic correlation (−0.46), which therefore is of a strikingly lower magnitude than the strong genetic correlation (−0.75) (cf. eq. (1)). Our findings thus mirror the conclusion based on meta-analysis by Dochtermann (2011): the genetic and phenotypic correlations agree in sign, but individual genetic correlations may vary in magnitude from the phenotypic ones. The extent by which the difference in magnitude, rather than in sign, of (one of) the genetic correlations affect the evolutionary trajectory of personality traits depends on the strength and direction of the natural selection on the different personality traits (Lynch and Walsh 1998). It is, nevertheless, clear from the strong correlations in the **G** matrix that selection on one personality trait will lead to a strong correlated response of the others personality traits in the direction more (less) aggressive/lower (higher) breathing rate/less (more) docile.

## Conclusions

Research on animal personality is currently primarily based on phenotypic measures of personality traits and phenotypic correlations between these (Dochtermann and Roff 2010; van Oers and Sinn 2011). While phenotypic-level analyses allow for valuable insights, they have restricted relevance for answering evolutionary questions. For example, we need to first ascertain that focal traits are heritable and that phenotypic correlations have a genetic basis in order to properly understand whether any selection on these traits is of evolutionary consequence (Grafen 1984). From this perspective, our study of nestling personality traits flags a promising avenue of research in animal personality. We find that field-based assays of nestling personality traits indeed can capture a genetic signal, both in terms of estimating heritability and in terms of estimating statistically significant genetic correlations between personality traits. Working with offspring facilitates obtaining the large sample sizes required for quantitative genetic estimates to have reasonably narrow confidence intervals. It also facilitates the implementation of an experimental design in a wild population, such as reciprocal cross-fostering, which further aids in estimation of quantitative genetic parameters. Knowledge of correlated personality in offspring opens up the possibility to study ontogenetic changes in behavioral syndromes (Stamps and Groothuis 2010) and allows to properly integrate natural selection into our understanding of how variation in animal personality is maintained in the wild.

## Supporting Information

**Figure S1.** Best Linear Unbiased Estimator (BLUE) of handling aggression with its approximate 95% confidence interval, as a function of measurement order. Measurement order reflect whether a nestling was measured first (order 1) in its brood or whether it had to spent additional time in isolation before being measured. Approximate 95% confidence intervals are double the standard error of the BLUE. Measurement order 1 is the model's intercept and the other BLUEs are calculated relative to this value as a factorial fixed effect (see Table 1).

**Table S1**. Variance-covariance matrix and correlation between three offpsring personality traits: handling aggression (AGG), breathing rate (BR), and docility (DOC). Variances in the diagonal, covariances below the diagonal. The correlation (printed in bold) above the diagonal are the same as given in Table 2. REML phenotypic estimates are the sum of its components. Because the nest-of-origin variance for handling aggression was constrained to zero, some of the covariances are not estimable (n.e.).

**Table S2.** Comparison of heritability (*h*^2^) estimated using the animal model, where the additive genetic effects are modeled explicitly, compared with heritability of a model only based on reciprocal cross-fostering. Animal model heritability is as given in Table 1. Cross-foster heritability is based on the same data, but assumes that twice the nest-of-origin variance estimates the additive genetic variance. The inflation of using only the cross-fostered data is calculated as cross-foster *h*^2^ divided by animal model *h*^2^ where a value larger than 1 indicates that ignoring the pedigree structure inflates the estimate of heritability. The difference in heritability estimates are not statistically significant, as judged using a *t*-test on the estimates, and their difference should be interpreted as illustration only.

## References

[b1] Adams MJ, King JE, Weiss A (2012). The majority of genetic variation in Orangutan personality and subjective well-being is nonadditive. Behav. Genet.

[b2] Baugh AT, Schaper SV, Hau M, Cockrem JF, de Goede P, van Oers K (2012). Corticosterone responses differ between lines of great tits (Parus major) selected for divergent personalities. Gen. Comp. Endocrinol.

[b3] Bell AM, Hankison SJ, Laskowski KL (2009). The repeatability of behaviour: a meta-analysis. Anim. Behav.

[b4] Both C, Dingemanse NJ, Drent PJ, Tinbergen JM (2005). Pairs of extreme avian personalities have highest reproductive success. J. Anim. Ecol.

[b5] Brommer JE, Alho JS, Biard C, Chapman JR, Charmantier A, Dreiss A (2010). Passerine extra-pair mating dynamics: a Bayesian model comparison of four species. Am. Nat.

[b6] Brommer JE, Pitala N, Siitari H, Kluen E, Gustafsson L (2011). Body size and immune defence of blue tit nestlings in response to manipulation of ectoparasites and food supply. Auk.

[b7] Carere C, van Oers K (2004). Shy and bold great tits (Parus major): body temperature and breath rate in response to handling stress. Physiol. Behav.

[b8] Charmantier A, Réale D (2005). How do misassigned paternities affect the estimation of heritability in the wild?. Mol. Ecol.

[b9] Cheverud JM (1988). A comparison of genetic and phenotypic correlations. Evolution.

[b10] Cockrem J (2007). Stress, corticosterone responses and avian personalities. J. Ornithol.

[b12] Crnokrak P, Roff DA (1995). Dominance variance: associations with selection and fitness. Heredity.

[b13] Dochtermann NA (2011). Testing Cheverud's conjecture for behavioral correlations and behavioral syndromes. Evolution.

[b14] Dochtermann NA, Roff DA (2010). Applying a quantitative genetics framework to behavioural syndrome research. Philos. Trans. R. Soc. B Biol. Sci.

[b15] Duckworth R, Kruuk LEB (2009). Evolution of genetic integration between dispersal and colonization ability in a bird. Evolution.

[b16] Elphinstone MS, Hinten GN, Anderson MJ, Nock CJ (2003). An inexpensive and high-throughput procedure to extract and purify total genomic DNA for population studies. Mol. Ecol. Notes.

[b17] Falconer DS, MacKay TFC (1996). Introduction to quantitative genetics.

[b18] Fisher RA (1958). The genetical theory of natural selection.

[b19] Fucikova E, Drent PJ, Smits N, van Oers K (2009). Handling stress as a measurement of personality in great tit nestlings (Parus major). Ethology.

[b20] Grafen A, Krebs JR, Davies NB (1984). Natural selection, kin selection and group selection. Behavioural ecology: an evolutionary approach.

[b21] Griffiths R, Double MC, Orr K, Dawson RJG (1998). A DNA test to sex most birds. Mol. Ecol.

[b22] Hessing MJC, Hagelsø AM, Wiepkema JAM, van Beek PR, Schouten WGP, Krukow R (1993). Individual behavioural characteristics in pigs. Appl. Anim. Behav. Sci.

[b23] Kenward MG, Roger JH (1997). The precision of fixed effects estimate from restricted maximum likelihood. Biometrics.

[b24] Kluen E, Brommer M, de Heij JE (2011). Adjusting the timing of hatching to changing environmental conditions has fitness costs in blue tits. Behav. Ecol. Sociobiol.

[b26] Koolhaas JM, Korte SM, Hopster SF, De Boer BJ, Van Der Vegt CG, Van Reenen H (1999). Coping styles in animals: current status in behaviour and stress-physiology. Neurosci. Biobehav. Rev.

[b27] Krebs JR, Davies NB (1978). Behavioural ecology: an evolutionary approach.

[b28] Kruuk LEB (2004). Estimating genetic parameters using the ‘animal model’. Philos. Trans. R. Soc. B Biol. Sci.

[b29] Kruuk LEB, Hadfield J (2007). How to separate genetic and environmental causes of similarity between relatives. J. Evol. Biol.

[b30] Kruuk LEB, Merilä J, Sheldon BC (2001). Phenotypic selection on heritable size traits revisited. Am. Nat.

[b31] Likert R (1932). A technique for the measurement of attitudes. Arch. Psychol.

[b32] Lynch M, Walsh B (1998). Genetics and analysis of quantitative traits.

[b33] Martins TLF, Roberts ML, Giblin I, Huxham R, Evans MR (2007). Speed of exploration and risk-taking behavior are linked to corticosterone titres in zebra finches. Horm. Behav.

[b34] Merilä J, Kruuk LEB, Sheldon BC (2001). Natural selection on the genetical component of variance in body condition in a wild bird population. J. Evol. Biol.

[b35] Naguib M, Flörcke C, van Oers K (2011). Effects of social conditions during early development on stress response and personality traits in great tits (*Parus major*. Dev. Psychobiol.

[b36] van Noordwijk AJ, de Jong G (1986). Acquisition and allocation of resources: their influence on variation in life history tactics. Am. Nat.

[b37] van Oers K, Sinn DL, Inoue-Murayama M, Kawamura S, Weiss A (2011). Toward a basis for the phenotypic gambit: advances in the evolutionary genetics of animal personality. From genes to animal behavior, primatology monographs.

[b38] van Oers K, Drent PJ, van Noordwijk G, de Jong AJ (2004). Additive and nonadditive genetic variation in avian personality traits. Heredity.

[b39] van Oers K, Kempenaers G, de Jong AJ, van Noordwijk B, Drent PJ (2005). Contribution of genetics to the study of animal personalities: a review of case studies. Behaviour.

[b40] Pitala N, Gustafsson L, Sendecka J, Brommer JE (2007). Nestling immune response to phytohaemagglutinin is not heritable in collared flycatchers. Biol. Lett.

[b41] Pitala N, Siitari H, Gustafsson L, Brommer JE (2009). Ectoparasites help to maintain variation in cell-mediated immunity in the blue tit–hen flea system. Evol. Ecol. Res.

[b42] Réale D, Gallant BY, Leblanc M, Festa-Bianchet M (2000). Consistency of temperament in bighorn ewes and correlates with behaviour and life history. Anim. Behav.

[b43] Réale D, Reader SM, Sol D, McDougall P, Dingemanse NJ (2007). Integrating temperament in ecology and evolutionary biology. Biol. Rev.

[b44] Réale D, Dingemanse NJ, Kazem AJN, Wright J (2010). Evolutionary and ecological approaches to the study of personality. Philos. Trans. R. Soc. B Biol. Sci.

[b45] Roff DA (1997). Evolutionary quantitative genetics.

[b46] Roff DA (2007). Evolutionary quantitative genetics.

[b47] Schuett W, Dall SRX, Royle NJ (2011). Pairs of zebra finches with similar ‘personalities’ make better parents. Anim. Behav.

[b48] Serenius T, Stalder KJ, Puonti M (2006). Impact of dominance effects on sow longevity. J. Anim. Breed. Genet.

[b49] Sih A, Bell A, Johnson JC (2004a). Behavioral syndromes: an ecological and evolutionary overview. Trends Ecol. Evol.

[b50] Sih A, Bell AM, Johnson JC, Ziemba RE (2004b). Behavioural syndromes: an integrative overview. Q. Rev. Biol.

[b51] Silverin B (1986). Corticosterone-binding proteins and behavioral effects of high plasma levels of corticosterone during the breeding period in the pied flycatcher. Gen. Comp. Endocrinol.

[b52] Silverin B (1998). Behavioural and hormonal responses of the pied flycatcher to environmental stressors. Anim. Behav.

[b53] Stamps JA, Groothuis TGG (2010). Ontogeny of animal personality: relevance, concepts and perspectives. Biol. Rev.

