# Supplementary figures and images for: Exploring the genetics of nestling personality traits in a wild passerine bird: testing the phenotypic gambit
Source: Ecol Evol. 2012 Nov 2;2(12):3032–44. doi: 10.1002/ece3.412 (PMC3538998; doi:10.1002/ece3.412)

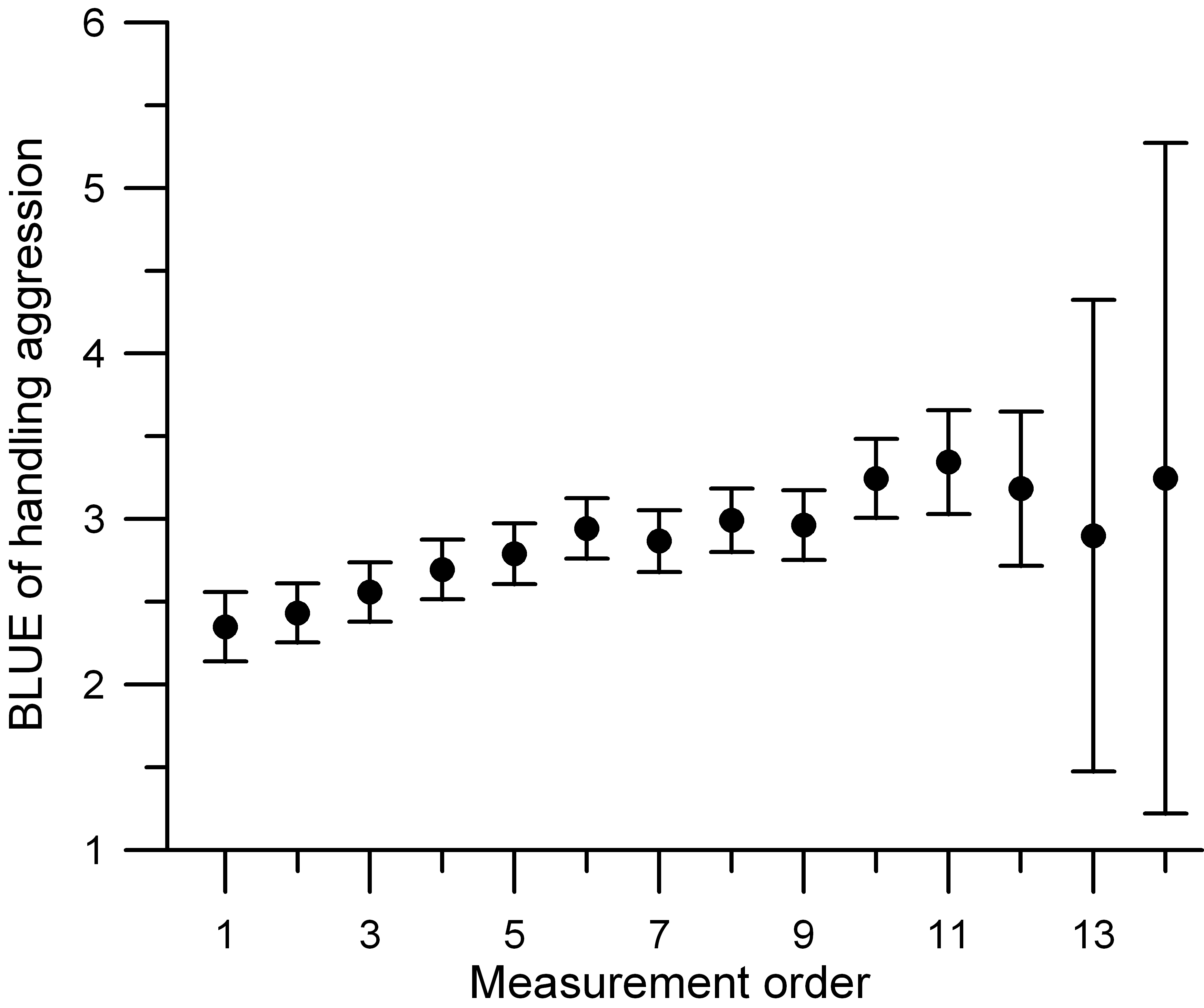

Supplement: Supplementary file 1 [file ece30002-3032-SD1.tif]
