# Supplementary material for: Exploring the genetics of nestling personality traits in a wild passerine bird: testing the phenotypic gambit
Source: Ecol Evol. 2012 Nov 2;2(12):3032–44. doi: 10.1002/ece3.412 (PMC3538998; doi:10.1002/ece3.412)
Supplement: Supplementary file 2 [file ece30002-3032-SD2.docx]

**Table S1**. Variance-covariance matrix and correlation between three offpsring personality traits: handling aggression (AGG), breathing rate (BR) and docility (DOC). Variances in the diagonal, covariances below the diagonal. The correlation (printed in bold) above the diagonal are the same as given in Table 2. REML phenotypic estimates are the sum of its components. Because the nest-of-origin variance for handling aggression was constrained to zero, some of the covariances are not estimable (n.e.).

**––––––––––––––––––––––––––––––––––––––––––––––––––––––––––––––––––––––––**

AGG BR DOC

––––––––––––––––––––––––––––––––––––––––––––––––––––––––––––––––––––––––

**REML phenotypic**

AGG 1.267 ± 0.048 **–0.278 ± 0.025 –0.463 ± 0.020**

BR –0.116 ± 0.012 0.139 ± 0.054 **0.372 ± 0.023**

DOC –0.0817 ± 0.0051 0.217 ± 0.0016 0.0245 ± 8.8E–3

**Residual**

AGG 0.735 ± 0.051 **–0.236 ± 0.044 –0.376 ± 0.037**

BR –0.574 ± 0.012 0.080 ± 0.006 **0.402 ± 0.041**

DOC –0.042 ± 0.0055 0.217 ± 0.0016 0.016 ± 0.001

**Nest-of-origin**

AGG 0 n.e. n.e.

BR n.e. 7.22E–3 ± 4.5E–3 **–1.63E–4 ± 1.2E–3**

DOC n.e. –0.16 ± 0.0012 5.41E–4 ± 5.8E–4

**Nest-of-rearing**

AGG 0.178 ± 0.033 **–0.138 ± 0.12 –0.458 ± 0.11**

BR –8.80E–3 ± 8.2E–3 0.023 ± 0.004 **0.286 ± 0.13**

DOC –9.84E–3 ± 3.4E–3 2.19E–3 ± 1.1E–3 2.58E–3 ± 5.8E–4

**Additive genetic (G)**

AGG 0.354 ± 0.072 **–0.503 ± 0.15 –0.747 ± 0.12**

BR –0.050 ± 0.018 0.028 ± 0.010 **0.429 ± 0.21**

DOC –0.030 ± 0.0077 4.89E–3 ± 2.8E–3 0.0046 ± 1.5E–3

**––––––––––––––––––––––––––––––––––––––––––––––––––––––––––––––––––––––––**

**Table S2.** Comparison of heritability (h^2^) estimated with the animal model, where the additive genetic effects are modelled explicitly, compared with heritability of a model only based on reciprocal cross-fostering. Animal model heritability is as given in Table 1. Cross-foster heritability is based on the same data, but assumes that twice the nest-of-origin variance estimates the additive genetic variance. The inflation of using only the cross-fostered data is calculated as cross-foster h^2^ divided by animal model h^2^ where a value larger than 1 indicates that ignoring the pedigree structure inflates the estimate of heritability. The difference in heritability estimates are not statistically significant, as judged by a t-test on the estimates, and their difference should be interpreted as illustration only.

**––––––––––––––––––––––––––––––––––––––––––––––––––––––––––––––––––––––––**

Trait animal model h^2^ cross foster h^2^ Inflation

––––––––––––––––––––––––––––––––––––––––––––––––––––––––––––––––––––––––

Aggression 0.285 ± 0.0540 0.252 ± 0.0499 0.88

Breating rate 0.171 ± 0.0763 0.307 ± 0.0527 1.8

Docility 0.159 ± 0.0664 0.238 ± 0.0485 1.5

––––––––––––––––––––––––––––––––––––––––––––––––––––––––––––––––––––––––
